# Rational metabolic-flow switching for the production of exogenous secondary metabolites in bamboo suspension cells

**DOI:** 10.1038/s41598-018-31566-4

**Published:** 2018-09-04

**Authors:** Taiji Nomura, Shinjiro Ogita, Yasuo Kato

**Affiliations:** 1 Biotechnology Research Center and Department of Biotechnology, Toyama Prefectural University, 5180 Kurokawa, Imizu, Toyama 939-0398 Japan; 2 Faculty of Life and Environmental Sciences, Prefectural University of Hiroshima, 5562 Nanatsukacho, Shobara, Hiroshima 727-0023 Japan

## Abstract

The synthetic biology-driven production of high-value plant secondary metabolites in microbial hosts has attracted extensive attention despite various challenges, including correct protein expression and limited supplies of starting materials. In contrast, plant cell cultures are rarely used for this purpose owing to their slow proliferation rates and laborious transformation processes. Here, we propose a “rational metabolic-flow switching” strategy to efficiently produce exogenous secondary metabolites using suspension-cultured bamboo (*Phyllostachys nigra*; Pn) cells as model production hosts. The Pn cells biosynthesise hydroxycinnamic acid amides (HCAAs) of putrescine as major secondary metabolites, which indicates that the phenylpropanoid and polyamine biosynthetic pathways are highly active and that the Pn cells may produce alternative secondary metabolites derived from those pathways. Stable transformants of Pn cells expressing agmatine coumaroyltransferase of barley (*Hordeum vulgare*) were generated with the expectation of metabolic-flow switching from HCAAs of putrescine to those of agmatine. In the recombinant Pn cells, the levels of HCAAs of putrescine decreased and the HCAAs of agmatine were produced instead. The production titre of the major product, *p*-coumaroylagmatine, reached approximately 360 mg/L, providing a proof-of-concept for the usefulness of “rational metabolic-flow switching” in synthetic biology using plant cell hosts.

## Introduction

Higher plants produce an estimated 200,000 or more secondary metabolites, also known as specialised metabolites or natural products^1–3^. Plant secondary metabolites have received considerable attention because they are sources of industrial and medicinal materials for human use, such as natural rubber, dyes, flavours and fragrances, spices, nutritional supplements, microbicides, insecticides, and pharmaceuticals^4,5^. Most commercialised high-value plant secondary metabolites are extracted from their native plant sources, semi-synthesised from extracted intermediates, or synthesised chemically. The major bottlenecks in the plant extraction process are the low yield and the complicated downstream purification processes. Although contemporary organic synthesis has enabled the production and modification of extremely complex natural products, it is not efficient and requires the use of toxic reagents, organic solvents, and extreme reaction conditions. Thus, the process is not amenable to large-scale production and does not meet the general industrial trend for ‘green’ processes having minimal environmental impacts^6,7^.

The establishment of other options for the preparation of high-value products has been explored, including improving existing plant sources through classical breeding, genetic engineering, and cell culturing^8^. In addition, over the past decade, the production of natural plant products in genetically tractable microbial hosts, such as *Escherichia coli* and *Saccharomyces cerevisiae*, has been performed. By reconstituting the biosynthetic pathways in the host microorganisms, terpenoids^9,10^, alkaloids^11–13^, and phenylpropanoids/polyketides^14–17^ have been produced. The most prominent milestone was the fermentative production of artemisinic acid, a precursor of antimalarial artemisinin, in an engineered *S. cerevisiae* strain with a surprisingly high production titre (up to 25 g/L)^10^. However, even if the target compound is successfully produced, its initial production titre is, in most cases, in the microgram to sub-milligram range per litre of culture, and substantial efforts are expended for the required thousand- to million-fold improvements in the productivity levels^18^.

The choice of an appropriate host system is crucial for the success of heterologous production^19^. *E. coli* is usually the simplest and cheapest expression system, but its use is limited owing to problems associated with correct protein folding and the lack of post-translational modifications. The lack of intracellular compartments often hampers the efficient expression of eukaryotic enzymes, such as endoplasmic reticulum membrane-bound cytochrome P450s, which are involved in the many aspects of plant secondary metabolite biosynthesis. Additionally, *E. coli* does not provide the endogenous precursors required for the biosynthesis of some classes of secondary metabolites, such as those originating from the mevalonate pathway that are needed for terpenoid biosynthesis^19^. Thus, the supply of the precursor compound to the culture medium or the introduction of enzymes for the biosynthesis of fundamental starting materials is requisite^11,18,20^. Although eukaryotic *S. cerevisiae* provides several distinct advantages over *E. coli*, such as having intracellular compartments, which allows for post-translational modifications, and the functional expression of membrane-bound enzymes, the glycosylation pattern is, in many cases, different from that in plants, which makes it sub-optimal. Additionally, the problems associated with supplying the fundamental starting material still need to be overcome^10^. When using microorganisms as production hosts, the negative and toxic effects of the supplied precursors and heterologously produced metabolites to the host organism must also be taken into consideration^19^.

While the heterologous production of plant secondary metabolites in microbial hosts has been extensively pursued, efforts to use plant suspension cells have not. Although there are several limitations to using plant cell cultures to produce secondary metabolites in comparison with using microorganisms, including slow proliferation rates and laborious transformation processes, plant cells are essentially the most suitable hosts for the heterologous expression of plant enzymes, and most of the obstacles associated with heterologous expression in microbial hosts can likely be overcome in plant cell cultures. One of the most significant challenges is the tendency of undifferentiated cells to accumulate secondary metabolites to a lesser extent and sometimes not at all, or to synthesise secondary metabolites distinct from those in the mother plant, depending on the culture conditions^19,21,22^. Thus, recovering or surpassing the biosynthetic levels of desired compounds inherent in the mother plant is required, as exemplified by the optimisation of a multitude of parameters affecting shikonin production in *Lithospermum erythrorhizon* cell suspension cultures^23^. When a cell culture of interest produces a compound distinct from the target compound, the culture system is likely to be excluded from further applications. When a certain secondary metabolic pathway is highly active in the cells, this indicates that the cells are promising production hosts for exogenous secondary metabolites derived from that active endogenous biosynthetic pathway. This can be achieved by introducing the exogenous biosynthetic gene(s) through genetic transformation. Because the plant cells should be excellent hosts for exogenous gene expression, this concept expands the range of applications of plant cell cultures in high-value metabolite production.

**Figure 1:**
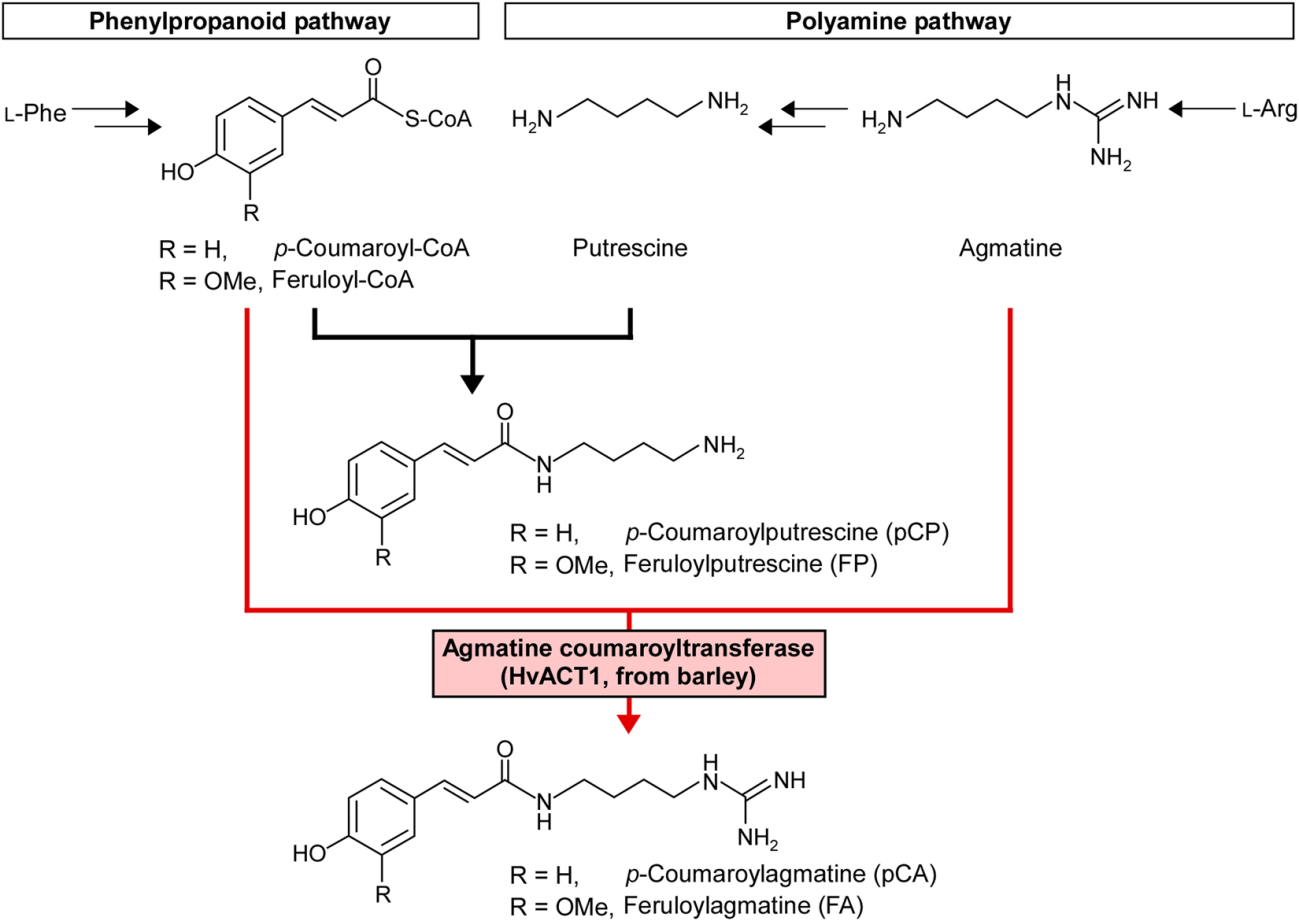
Metabolic-flow switching of bamboo Pn cells. The *HvACT1* gene of barley that encodes agmatine coumaroyltransferase (ACT) was introduced into Pn cells to switch the biosynthetic pathway from producing hydroxycinnamic acid amides (HCAAs) of putrescine to producing those of agmatine.

To prove this concept, we demonstrate efficient metabolic engineering using previously established bamboo cells as a model system. We have created an efficient callus and suspension cell culture system for the bamboo *Phyllostachys nigra* (Pn) and determined the culture conditions that promoted a high degree of lignification (two lignification conditions; LG1 and LG2) or rapid proliferation without lignin deposition (proliferation condition; PR)^24–26^. The Pn cells cultured under LG1 and LG2 conditions accumulated feruloylputrescine (FP) as major secondary metabolite accompanied by a smaller amount of *p*-coumaroylputrescine (pCP) (Fig. 1), but these compounds were scarcely accumulated under the PR conditions^27^. Hydroxycinnamic acid amides (HCAAs), like FP and pCP, are widely distributed in the plant kingdom, and they are biosynthesised through the *N*-coupling reaction of (poly)amines to hydroxycinnamoyl-CoAs, which is catalysed by a specific acyltransferase^28^. Its presence indicates that feruloyl-CoA and putrescine are being actively synthesised in the Pn cells cultured under LG1 and LG2 conditions. In this study, we transformed Pn cells with agmatine coumaroyltransferase (ACT) of barley (*Hordeum vulgare*)^29^, which has a primary function of forming *p*-coumaroylagmatine (pCA) from *p*-coumaroyl-CoA and agmatine, intermediates for feruloyl-CoA and putrescine, respectively, to determine whether an efficient switching of the metabolic flow from HCAAs of putrescine to those of agmatine was possible by using the intermediates for FP biosynthesis as substrates for the ACT enzyme in the recombinant Pn cells (Fig. 1). Based on this proof-of-concept study, the foreseeable contribution of such “rational metabolic-flow switching” to expand the use of plant cell cultures as hosts for the production of exogenous high-value secondary metabolites is described.

## Methods

### Cell cultures

Pn suspension cells^24^, which are currently available from the RIKEN Bioresource Center (no. rpc00047; http://ja.brc.riken.jp/), were maintained in modified Murashige and Skoog (MS) liquid medium^30^ supplemented with 680 mg/L KH_2_PO_4_, 10 μM 4-amino-3,5,6-trichloropyridine-2-carboxylic acid (Picloram), and 3% (w/v) sucrose. This medium strongly promotes the proliferation of Pn cells^25^ and is referred to as the PR conditions. The cells were subcultured in 100 mL liquid medium in a 300-mL Erlenmeyer flask and maintained on a rotary shaker (110 rpm) in the dark at 25 °C. To maintain stable morphology and synchronous growth, the cells were subcultured every two weeks by adjusting the initial sedimented cell volume (SCV) to 2.5% as described previously^25^. To promote lignification, as well as FP/pCP biosynthesis, in the cells, 2-week-old cells cultured under PR conditions were transferred to the following fresh liquid media: half-strength MS medium (1/2 MS) containing 3% (w/v) sucrose (LG1 conditions) and 1/2 MS medium supplemented with 10 μM 6-benzyladenine (BA) and 3% (w/v) sucrose (LG2 conditions)^26,27^. They were cultured as described above. Pn callus cells^24^ were maintained on PR medium solidified with 0.3% (w/v) gellan gum in a Petri dish (φ = 90 mm). The cultures were incubated in the dark at 25 °C, and the subculturing was carried out at approximately 4-week intervals by transferring the calli [approximately 100 mg fresh weight (FW)] to the fresh medium.

### Generation of stable transformants expressing the barley HvACT1 gene

The pBIH1-IG vector^31^, carrying the hygromycin phosphotransferase (*HPT*) and neomycin phosphotransferase II (*NPT II*) genes as the selectable markers, was used as the backbone for the transformation vector. The original intron–GUS sequence of the vector, which is located between the cauliflower mosaic virus (CaMV) 35S promoter and the nopaline synthase terminator, was cut out by restriction digestion with XbaI and SacI, and the vector was blunt-ended with T_4_ DNA polymerase (Takara Bio, Shiga, Japan), followed by 5′-dephosphorylation with a calf intestine alkaline phosphatase (Takara Bio). Full-length cDNA encoding ACT of barley (*H. vulgare*; GenBank accession no. AB334132) was amplified by PCR using the pUC118 vector harbouring *HvACT1* cDNA^32^ as the template, 5′-phosphorylated with a T_4_ polynucleotide kinase (Takara Bio), and ligated to the blunt-ended vector to generate the transformation vector pBIH1-HvACT1.

The transformation of the Pn cells with pBIH1-HvACT1 was performed by particle bombardment using the Biolistic Particle Delivery System (PDS-1000/He, Bio-Rad, Hercules, CA, USA) as described previously^25^. After bombardment, the calli were grown for 1–2 weeks on a solid PR medium without hygromycin B, and then transferred to a selective medium (solid PR medium supplemented with 100 mg/L hygromycin B). After several rounds of subculturing on a selective medium at 2- to 4-week intervals, the hygromycin B-resistant cell lines were established. They were maintained on a solid PR medium supplemented with 100 mg/L hygromycin B and in a liquid PR medium without hygromycin B as suspension cells.

### Genomic and RT-PCR analyses

Genomic DNA was purified from hygromycin B-resistant cells, as well as from the wild-type Pn cells, using a DNeasy Plant Mini Kit (Qiagen, Venlo, Netherlands), and subjected to PCR analyses of *HvACT1*, *HPT*, and actin genes. For the RT-PCR analysis, total RNA was purified from hygromycin B-resistant and genomic PCR-positive cells, as well as from the wild-type Pn cells, using an RNeasy Plant Mini Kit (Qiagen), followed by a DNase I treatment. The cDNA was synthesised using the SuperScript III First-Strand Synthesis System (Invitrogen, Carlsbad, CA, USA) and subjected to PCR analysis. Detailed PCR conditions are described in the Supplementary Methods.

### Immunoblot analysis

The rabbit anti-HvACT1 polyclonal antibody was prepared by a professional service (BioGate, Gifu, Japan) using the His-tag-free recombinant HvACT1 enzyme (see below “Expression and purification of recombinant HvACT1 enzyme”) as the antigen. Aliquots of the serum were subjected to a Melon Gel IgG Purification Kit (Thermo Scientific, Waltham, MA, USA) to purify the IgG fraction.

Approximately 100–150 mg FW of the suspension cells was frozen in liquid nitrogen before being ground to a fine powder with a mortar and pestle. The powder was then extracted with 1.5 mL of 50 mM Tris-HCl buffer (pH 7.5), and the resulting extract was centrifuged at 15,000 × *g* for 20 min at 4 °C. The supernatant containing 2 μg of protein was subjected to SDS-PAGE with a 10% gel, and the proteins were transferred electrophoretically onto a polyvinylidene difluoride membrane (0.22 μm, ATTO, Tokyo, Japan). After blocking in Tris-buffered saline containing 1% (w/v) bovine serum albumin, reactions with the purified anti-HvACT1 IgG (5 μg in 4-mL reaction solution) and then with the goat anti-rabbit IgG conjugated with alkaline phosphatase (Cell Signaling Technology, Danvers, MA, USA; 1 μL in 4-mL reaction solution) were performed in XL-Enhancer solution (APRO Life Science Institute, Tokushima, Japan), followed by signal detection using a 1-step NBT/BCIP kit (Thermo Scientific).

### Chemicals

FP and pCP were prepared by chemical synthesis as described previously^27^. Feruloylagmatine (FA) and pCA were synthesised according to the method described by Negrel and Smith^33^. Feruloyl- and *p*-coumaroyl-CoAs were synthesised as described by Stöckigt and Zenk^34^ by ester exchange via *N*-hydroxysuccinimide esters of ferulic- and *p*-coumaric acids, respectively.

### Analysis of HCAAs in cell extracts

Suspension cells cultured under PR, LG1, and LG2 conditions were extracted and analysed by HPLC according to Nomura *et al*.^27^ using synthetic FP, pCP, FA, and pCA as standards.

### Crude enzyme extraction and the enzyme assay

Approximately 500 mg FW of the suspension cells were frozen in liquid nitrogen before being ground to a fine powder with a mortar and pestle. The powder was then extracted with 3 mL of 50 mM Tris-HCl buffer (pH 7.5), and the resulting extract was centrifuged at 15,000 × *g* for 20 min at 4 °C. The supernatant was collected and passed through a PD-10 column (GE Healthcare, Piscataway, NJ, USA), which had been equilibrated with the same buffer, and the eluent was used as the crude enzyme to measure the ACT activity. Protein concentrations were determined with a Protein Assay Kit (Bio-Rad) using a bovine serum albumin standard.

Standard enzyme reactions were performed in 50 mM Tris-HCl buffer (pH 7.5) containing 100 μL of crude enzyme, 100 μM *p*-coumaroyl-CoA or feruloyl-CoA, and 100 μM agmatine sulphate in a total volume of 200 μL. After incubation at room temperature for 20 min, the reaction was terminated by adding 20 μL of 1 N HCl, and the reaction products were quantified by HPLC analysis [column, TSKgel ODS-100V, 4.6 × 150 mm, 5 μm (Tosoh, Tokyo, Japan); solvent, 14% (v/v) acetonitrile in 0.1% (v/v) trifluoroacetic acid; flow rate, 0.8 mL/min; detection, 280 nm; column temperature, 35 °C].

### Time-course analyses of the HCAA content and expression profile of HvACT1

Subcultured suspension cells of wild-type Pn and ACT-transformants were transferred to each of the fresh PR, LG1, and LG2 media with initial cell density levels of 2.5% SCV, cultured, and collected as described previously^27^. The cells, which were collected every other day until 16 d, were subjected to the analyses of the following: (1) HCAA contents by HPLC, (2) *HvACT1* transcripts by RT-PCR, (3) HvACT1 protein by immunodetection, and (4) ACT activity levels in the crude extracts by the enzyme assay, all of which were performed as described above.

### Expression and purification of recombinant HvACT1 enzyme

The recombinant HvACT1 enzyme was expressed in a host/vector system of BL21-CodonPlus (DE3)-RIL/pET28a, and the His-tag-free recombinant enzyme was purified. Details are described in the Supplementary Methods.

### Recombinant enzyme characterisation

The molecular masses of the denatured and native forms of the enzyme were estimated by SDS-PAGE with 12.5% gel and gel-filtration analyses, respectively. The gel-filtration analysis was performed on a TSKgel G3000SWxl column (7.8 × 300 mm, 5 μm, Tosoh) equilibrated with 50 mM Tris-HCl buffer (pH 7.5) containing 150 mM NaCl using MW-Marker (HPLC) (Oriental Yeast, Tokyo, Japan) as the molecular size marker. Enzyme reactions for the determination of kinetic parameters were performed at room temperature in 50 mM Tris-HCl buffer (pH 7.5), containing 10 μL of appropriately diluted enzyme and varied concentrations of the acyl-donor and acyl-acceptor, in a total volume of 200 μL. To determine the kinetic parameters for the acyl-donor, *p*-coumaroyl-CoA and feruloyl-CoA concentrations were varied in the 0.5–10 μM and 0.5–20 μM ranges, respectively, with 100 μM agmatine sulphate as the acyl-acceptor. To determine the kinetic parameters for the acyl-acceptor, agmatine sulphate and putrescine dihydrochloride concentrations were varied in the 0.5–10 μM and 0.5–30 mM ranges, respectively, with 100 μM *p*-coumaroyl-CoA as the acyl-donor. The enzyme amount and the reaction time were adjusted to allow the reaction to proceed linearly, and the reaction termination and quantification of products were performed as described above. Kinetic parameters were calculated by nonlinear fitting of the data to the Michaelis–Menten equation using SigmaPlot 12.5 (Systat Software, San Jose, CA, USA).

## Results

### Enzymatic property of HvACT1

The gene encoding ACT was first identified in barley by Burhenne *et al*.^29^, and later Nomura *et al*.^32^ reported the isolation, from a different cultivar of barley, of its highly homologous sequence, designated *HvACT1*, which has a polypeptide sequence that differs by three amino acids from the original sequence. Because the enzymatic property of HvACT1 had not been examined, we enzymatically characterised the recombinant HvACT1 expressed in *E. coli* prior to the transformation of the bamboo Pn cells with the *HvACT1* gene. The recombinant HvACT1 enzyme was efficiently expressed in a soluble protein fraction in *E. coli*. The His-tag-free enzyme was purified to homogeneity through metal-affinity chromatography, followed by the removal of His-tag by thrombin digestion and gel-filtration chromatography. The yield was approximately 2 mg from 500 mL of *E. coli* culture. The purified enzyme appeared as a 49-kDa band by SDS-PAGE (Supplementary Fig. S1), and the native molecular mass was estimated to be 56 kDa by gel-filtration. These results confirmed that the recombinant enzyme exists as a monomer as reported previously^29^.

The specific activities of the recombinant HvACT1 enzyme for the formation of pCA and FA were 870 nkat/mg and 480 nkat/mg, respectively, while those for the formation of pCP and FP were 3.3 nkat/mg and 1.0 nkat/mg, respectively (Supplementary Table S1). This indicated that the enzyme prefers *p*-coumaroyl-CoA over feruloyl-CoA as the acyl-donor and agmatine over putrescine as the acyl-acceptor. Apparent *K*_m_ and *k*_cat_ values of the enzyme for acyl-donors and acyl-acceptors were determined when either the acyl-donor or acyl-acceptor concentration was fixed (Table 1, Supplementary Fig. S2). In the presence of 100 μM agmatine as the acyl-acceptor, the *K*_m_ and *k*_cat_ values for *p*-coumaroyl-CoA (1.1 μM and 39 s^−1^, respectively) were approximately twofold lower and higher, respectively, than those for feruloyl-CoA (2.0 μM and 18 s^−1^, respectively), resulting in an approximately fourfold higher catalytic efficiency (*k*_cat_/*K*_m_) for *p*-coumaroyl-CoA than for feruloyl-CoA. In the presence of 100 μM *p*-coumaroyl-CoA as the acyl-donor, the *K*_m_ value for agmatine (1.7 μM) was considerably lower than that for putrescine (7,200 μM), with the *k*_cat_ value being approximately eightfold higher for agmatine (38 s^−1^) than for putrescine (4.7 s^−1^). Thus, it was demonstrated that the HvACT1 enzyme favours *p*-coumaroyl-CoA over feruloyl-CoA as the acyl-donor and agmatine over putrescine as the acyl-acceptor, and that the enzyme recognises the structure of amine (acyl-acceptor) more rigorously than that of hydroxycinnamoyl-CoA (acyl-donor). Based on the substrate specificities of the HvACT1 enzyme, we confidently predicted that the transgenic Pn cells expressing HvACT1 would predominantly produce pCA.

**Table 1:** Kinetic parameters of the recombinant HvACT1 enzyme.

Substrate	*K*_m_ (μM)	*k*_cat_ (s^−1^)	*k*_cat_/*K*_m_ (s^−1^/μM)	Relative efficiency (%)
For acyl-donor				
*p*-Coumaroyl-CoA^a^	1.1 ± 0.12	39 ± 1.0	35	100
Feruloyl-CoA^a^	2.0 ± 0.14	18 ± 0.31	9.0	26
For acyl-acceptor				
Agmatine^b^	1.7 ± 0.13	38 ± 0.87	22	100
Putrescine^b^	7,200 ± 530	4.7 ± 0.12	0.00065	0.0030

Data are means ± SEs (SE: fitting error to the Michaelis–Menten equation as calculated by a nonlinear fitting program).

^a^At 100 μM agmatine as the acyl-acceptor.

^b^At 100 μM *p*-coumaroyl-CoA as the acyl-donor.

See Supplementary Fig. S2 for Michaelis–Menten plot of each analysis.

### Generation of stably transformed Pn cells expressing the HvACT1 gene

Pn cells were bombarded with the pBIH1-HvACT1 vector to express *HvACT1*, as well as *HPT*, as selectable marker. Of approximately 100 calli screened with the hygromycin B supplemented medium, 10 calli that survived in the selective medium were used as potential transformants, and the HvACT1 products pCA and FA were analysed in the cell extracts. As shown in Supplementary Fig. S3, six lines (2, 3, 8, 15, 22, and 31) were found to accumulate pCA and FA. pCA was the major product and only a small amount of FA was detected in each line. Neither pCA nor FA was detected in the other four lines (13, 17, 24, and 32), as well as in the wild-type Pn cells. Of the six pCA/FA-positive calli, the proliferation rates of lines 2, 3, 8, and 31 were very slow, while those of lines 15 and 22 were as fast as the wild-type rate. Therefore, lines 15 and 22, as elite lines, were further characterised.

**Figure 2:**
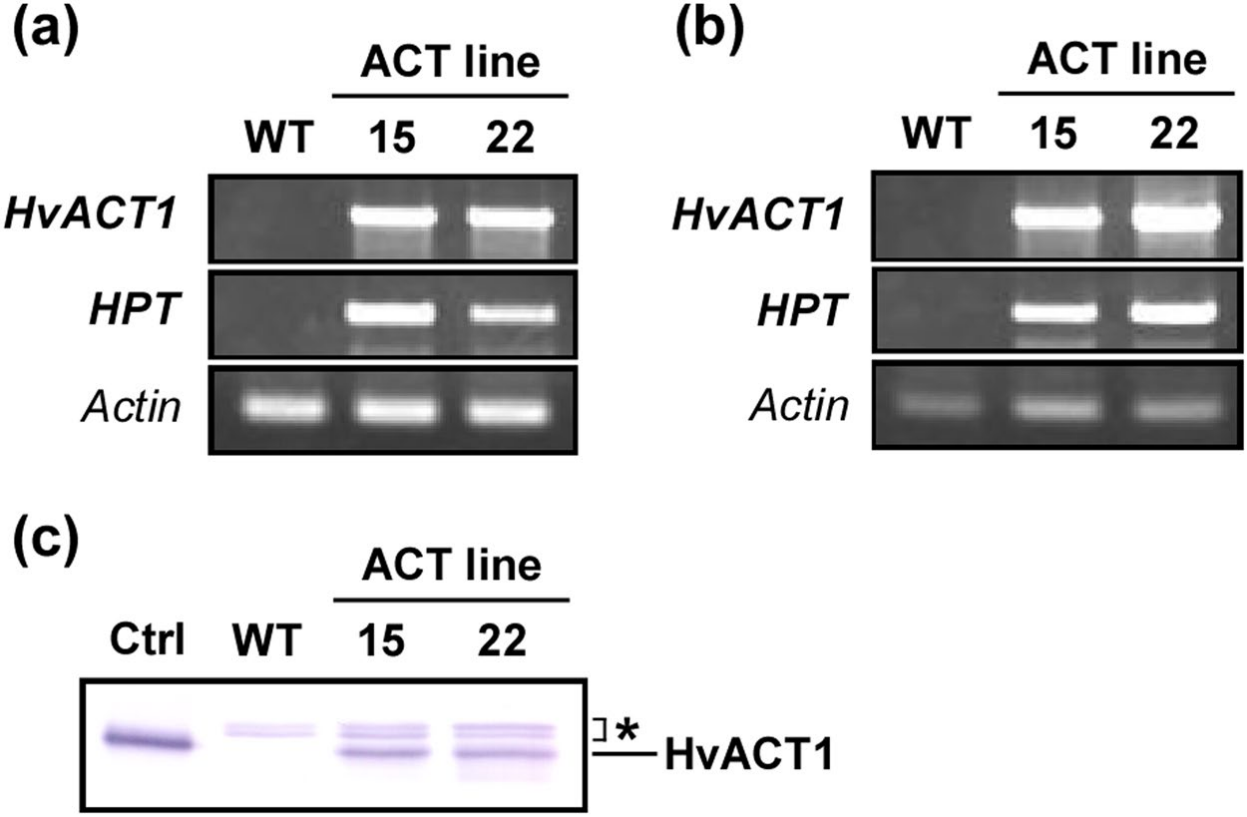
Genomic PCR, RT-PCR, and immunoblot analyses of ACT transformants. Eight-day-old suspension cells of wild-type (WT) Pn and ACT transgenic lines (15 and 22) cultured under PR conditions were analysed. For the PCR experiments (**a**,**b**), *HvACT1* and *HPT* transgenes were amplified to verify their stable maintenance using genomic PCR (**a**) and transcription using RT-PCR (**b**) and *actin* was amplified as an endogenous standard. Full-length gels for genomic PCR and RT-PCR analyses are presented in Supplementary Fig. S5a,b, respectively. For the immunoblot analysis (**c**) the recombinant His-tag-free HvACT1 enzyme expressed in *E. coli* was used as a positive control (Ctrl) for the detection of the HvACT1 protein in the crude cell extracts. Asterisks indicate that the signals occurred by a non-specific cross-reaction of the anti-HvACT1 polyclonal antibody with some protein(s) in the crude extracts. Full-length blot is presented in Supplementary Fig. S5c.

Genomic PCR verified that the *HvACT1* and the *HPT* transgenes were present and maintained stably in transgenic lines 15 and 22 (Fig. 2a). Both transgenes were transcribed, and the *HvACT1* transcripts were translated to protein, as determined by RT-PCR and immunoblot analyses, respectively (Fig. 2b,c).

The two ACT lines (15 and 22) were suspension cultured in a liquid medium. The suspension cells were cultured under three different conditions, PR, LG1, and LG2, and the changes in the cells’ growth profiles were monitored by measuring the SCV throughout the 16-d culture period. As shown in Supplementary Fig. S4, the SCVs of the two ACT lines reached approximately 80% under PR conditions and approximately 20–30% under LG1 and LG2 conditions. These profiles were comparable with those of wild-type Pn cells, indicating that the expression of the exogenous *HvACT1* gene had no negative effect on the growth profiles of the suspension cells.

### Expression profiles of HvACT1 in ACT transformants

**Figure 3:**
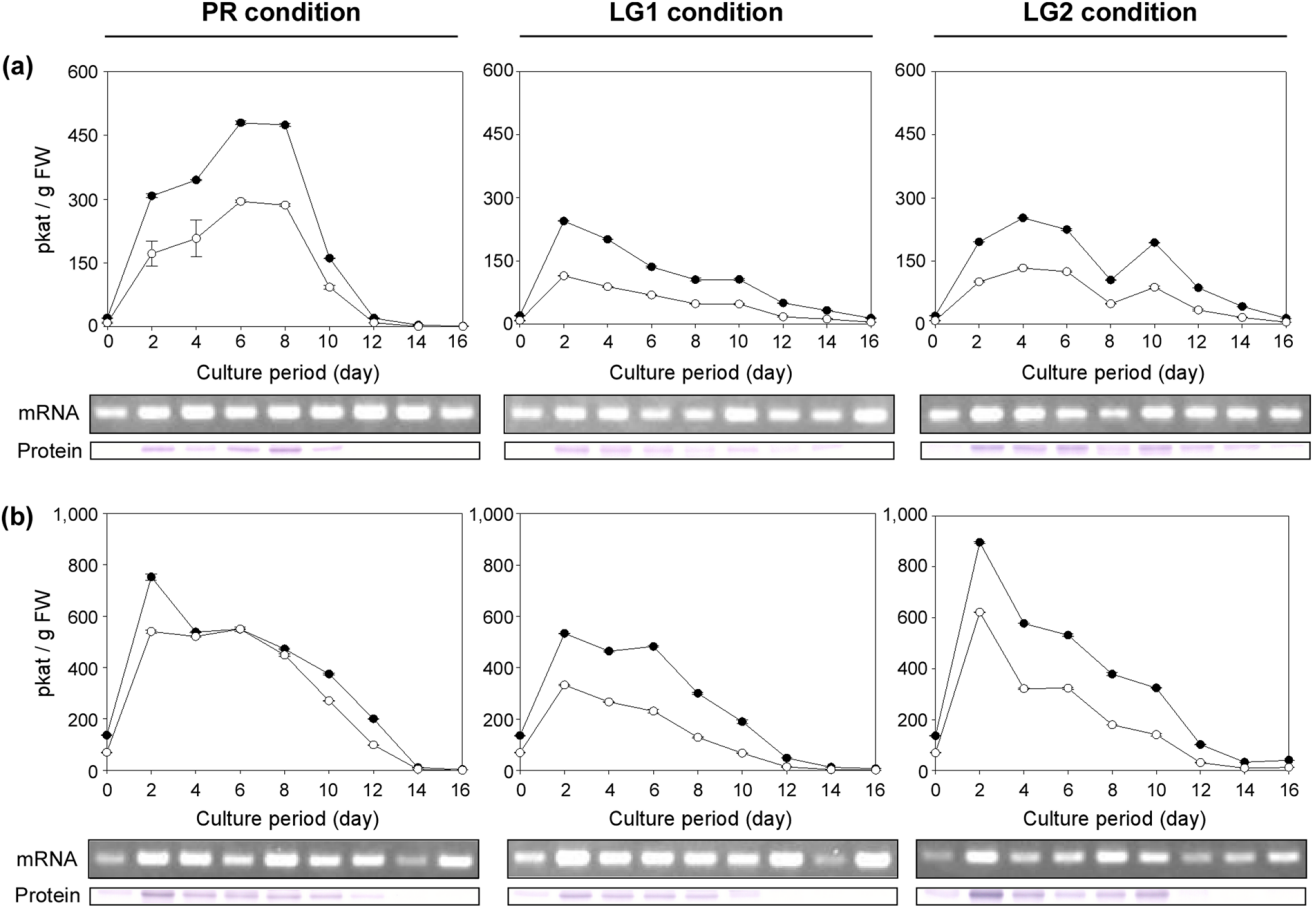
Time-course changes in ACT activities and *HvACT1* expression in ACT transformants cultured under PR, LG1, and LG2 conditions. ACT activities catalysing the formation of pCA (●) and FA (○) in the crude extracts were measured. *HvACT1* transcripts and HvACT1 protein were detected by RT-PCR (labelled mRNA) and immunoblot (labelled protein) analyses, respectively. (**a**) ACT-line 15 (**b**) ACT-line 22. Data are means ± SDs (*n* = 3). One katal (kat) of enzyme activity was defined as the amount of enzyme that catalyses the formation of the reaction product at a rate of 1 mol/s. Full-length gels for RT-PCR analysis and full-length blots for immunoblot analysis are presented in Supplementary Figs S6 and S7, respectively.

To determine whether the HvACT1 protein expressed in the ACT transformants (Fig. 2) functioned as an active enzyme, the ACT activities for the formation of pCA and FA were measured in the crude extracts of the suspension cells during the 16-d culture period. ACT activities were detected in the lines 15 and 22 (Fig. 3a,b) under PR, LG1, and LG2 conditions. No ACT activities were detected in the wild-type Pn cells under any culture conditions during the 16-d culture period, suggesting that Pn cells do not possess an endogenous enzyme catalysing the formation of the HCAAs of agmatine. In the ACT transformants, the pCA-forming activity was approximately twofold higher than the FA-forming activity, which coincided with the ratio of the specific activity as determined using the recombinant HvACT1 enzyme (Supplementary Table S1), indicating that HvACT1 was properly expressed in its active form in the Pn cells. The expression of the *HvACT1* gene was driven under the control of the CaMV35S promoter, allowing for a high level of constitutive transcription, and accordingly, the *HvACT1* transcripts were detected throughout the 16-d culture period (Fig. 3). However, the ACT activities in the crude extracts increased during the early culture stages and then decreased gradually to trace levels during the later culture stages. These patterns coincided with those of the translation products of HvACT1 as detected by the immunoblot analysis (Fig. 3). Thus, the supply of the HvACT1 enzyme is not constant because of the decrease in the protein synthesis rate during the later culture stages, when the cellular vitality is lowered, despite the use of the constitutive CaMV35S promoter.

### Changes in HCAA profiles in ACT transformants

**Figure 4:**
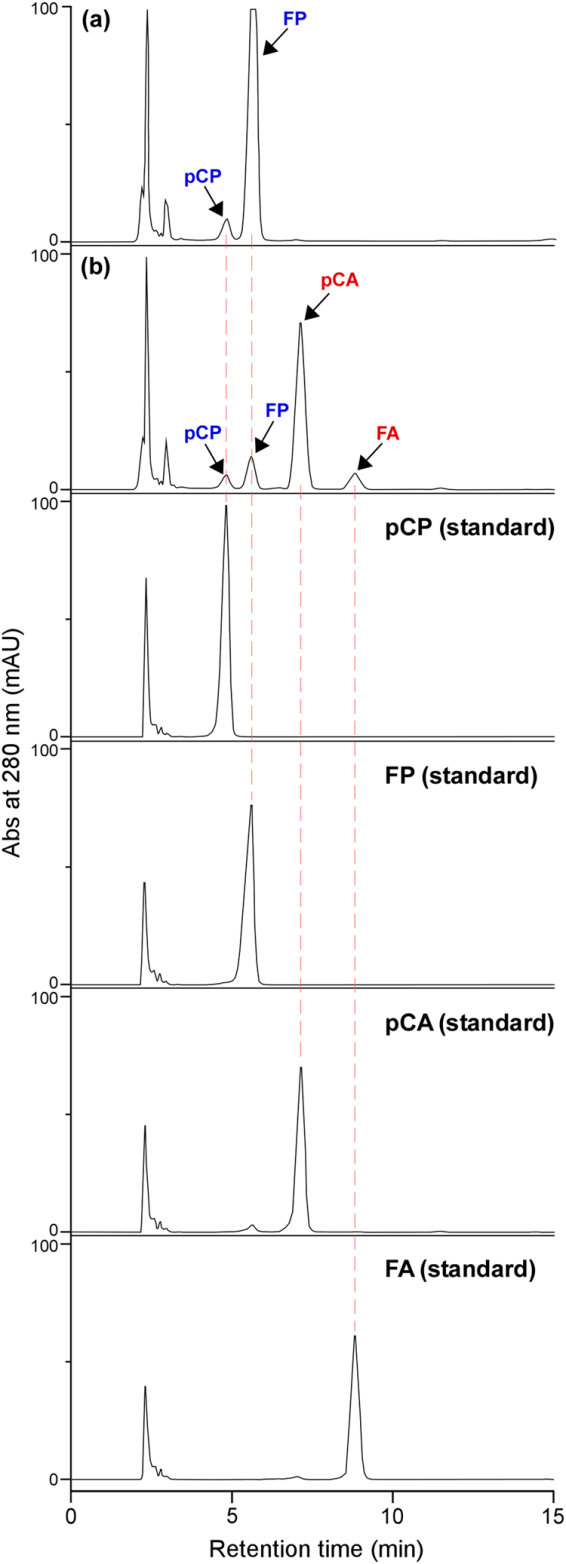
HPLC chromatograms representing the production of pCA and FA in ACT transformants. HPLC chromatograms of the extracts from 8-d-old suspension cells cultured under LG1 conditions are shown with chromatograms of the standard compounds (pCP, FP, pCA, and FA). (**a**) Wild-type (**b**) ACT transformant (ACT-line 22 as a representative).

**Figure 5:**
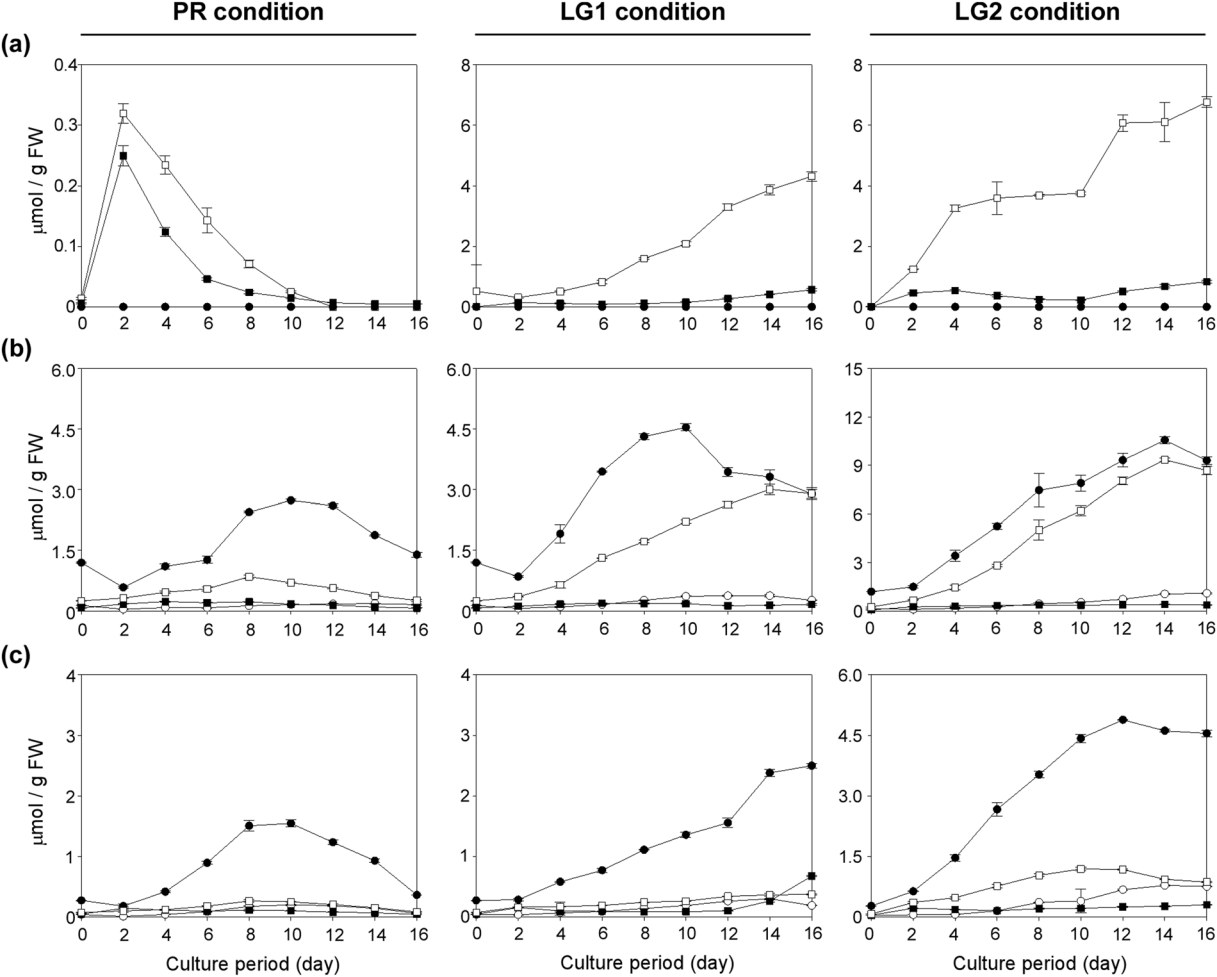
Time-course changes in HCAA contents in ACT transformants cultured under PR, LG1, and LG2 conditions. The contents of pCA (●), FA (○), pCP (■), and FP (□) in the suspension cells were measured. (**a**) Wild-type (**b**) ACT-line 15 (**c**) ACT-line 22. Data are means ± SDs (*n* = 3).

As we reported previously^27^, wild-type Pn suspension cells accumulate FP as a major secondary metabolite, which is accompanied by the accumulation of lesser amounts of pCP. Additionally, the FP content greatly increased under LG1 and LG2 conditions, while neither pCA nor FA was detected under any culture conditions (Figs 4a and 5a). The ACT transformants produced pCA instead of FP as the major HCAA amide (Figs 4b and 5b,c). The pCA contents increased as cells grew, and they reached maximal levels during the later culture stages. Afterward, the levels were kept constant or lowered gradually, which correlated with the expression profiles of the HvACT1 enzyme (Fig. 3). The retarded accumulation of pCA (Fig. 5b,c) as compared with the enzyme activity profile (Fig. 3) is probably because the supply of substrate, in particular *p*-coumaroyl-CoA, to the HvACT1 enzyme is highly activated during the later culture stages, which is associated with lignin biosynthesis in the cells cultured under LG1 and LG2 conditions^27^. The results indicate the replacement of FP biosynthesis with pCA biosynthesis in line 22 (Fig. 5c), suggesting that the HvACT1 enzyme expressed in the cells efficiently intercepted *p*-coumaroyl-CoA and agmatine in advance of their conversion to feruloyl-CoA and putrescine, respectively (Fig. 1). In line 15, however, not only pCA, but also FP, accumulated at levels comparable with those in the wild-type Pn cells (Fig. 5b). Because the pCA content in line 15 was greater than that in line 22, the HvACT1 enzyme in the former seems to be expressed more efficiently than in the latter. It is likely that the basal FP biosynthetic level in line 15 is greater than that in line 22, and thus, the effectiveness of the metabolic-flow switching from FP to pCA is diminished. Alternatively, putrescine may be being synthesised directly from ornithine, rather than through the pathway via agmatine^35,36^, in line 15. Recently, we identified an acyltransferase catalysing FP formation from feruloyl-CoA and putrescine in the Pn cells (unpublished data). The efficiency of the metabolic-flow switching from FP to pCA in the ACT transformants may be further improved by knocking out/down regulating the FP-forming acyltransferase using genome editing or RNA interference techniques.

By multiplying the pCA content by the cell FW at each point of collection, we calculated the production titre of pCA per litre of suspension culture. As a result, the highest titres in lines 15 and 22 were estimated to be 359 mg/L (at d-14 under LG2 conditions) and 173 mg/L (at d-16 under LG1 conditions), respectively. While on the other hand, the pCA concentrations in the culture medium were 1.6 mg/L (line 15 at d-14 under LG2 conditions) and 3.3 mg/L (line 22 at d-16 under LG1 conditions), which were much lower than those accumulated inside the cells, indicating that the pCA produced is hardly secreted into the culture medium due to hardening of cell walls by lignification.

## Discussion

In the present study, we successfully achieved the metabolic engineering of bamboo Pn cells. Introducing only one exogenous biosynthetic enzyme (HvACT1) allowed for the production of high levels of pCA, which is not present in the wild-type Pn cells. Here, we did not attempt to optimise the culture system for enhanced productivity, but the production titre achieved (up to 359 mg/L) was surprisingly high for an initial value. Although the subcellular localisation of pCA has not yet been elucidated, a large part of pCA produced is probably sequestered in a certain subcellular compartment (e.g. vacuole). Presence of a proper subcellular compartment (i.e. accumulation site) in plant cells provides a great advantage over the microbial hosts, especially as compared to prokaryotic *E. coli*, to reduce the cytotoxicity of the produced compound, leading to high productivity. To increase the productivity, many parameters have yet to be optimised, such as basal medium constituents, promoter sequences, and elicitors. Moreover, a part of pCA produced may probably be incorporated into cell walls, which is one of the common features of HCAAs, including pCA^37^. By controlling this feature, its production level may further increase. Optimising these factors that affect the productivity will provide a versatile system for the synthesis of exogenous high-value compounds, especially those having a phenylpropanoid moiety, using the Pn cells as the production host.

pCA serves as an antifungal agent^38–40^ and also as a direct precursor of the stronger antifungal agent, hordatine A, which is accumulated in barley^39,41^. Although the endogenous barley enzyme catalysing the dimerisation of pCA to form hordatine A has not yet been identified^32^, a radical coupling reaction catalysed by peroxidase has been reported to generate hordatine A *in vitro*^33,41,42^. Because this reaction can be reproduced by horseradish peroxidase (HRP) *in vitro*, introducing its encoding gene to the ACT transformed Pn cells would allow for the generation of a hordatine-producing system. Plant cell hosts would be more likely to functionally express and localise HRP compared with conventional microbial hosts because HRP undergoes post-translational modifications, including *N*-glycosylation at eight Asn residues, the formation of four disulphide bonds by eight Cys residues, haeme incorporation, Ca^2+^ incorporation, and vesicular transport to vacuoles^43–46^. The generation of stably transformed Pn cells expressing multiple exogenous genes is the next challenge that would make Pn cells versatile hosts capable of producing exogenous high-value compounds that are biosynthesised by multiple sequential enzymes. Thus, the generation of ACT–HRP double transformed Pn cells is now in progress in our laboratory.

**Figure 6:**
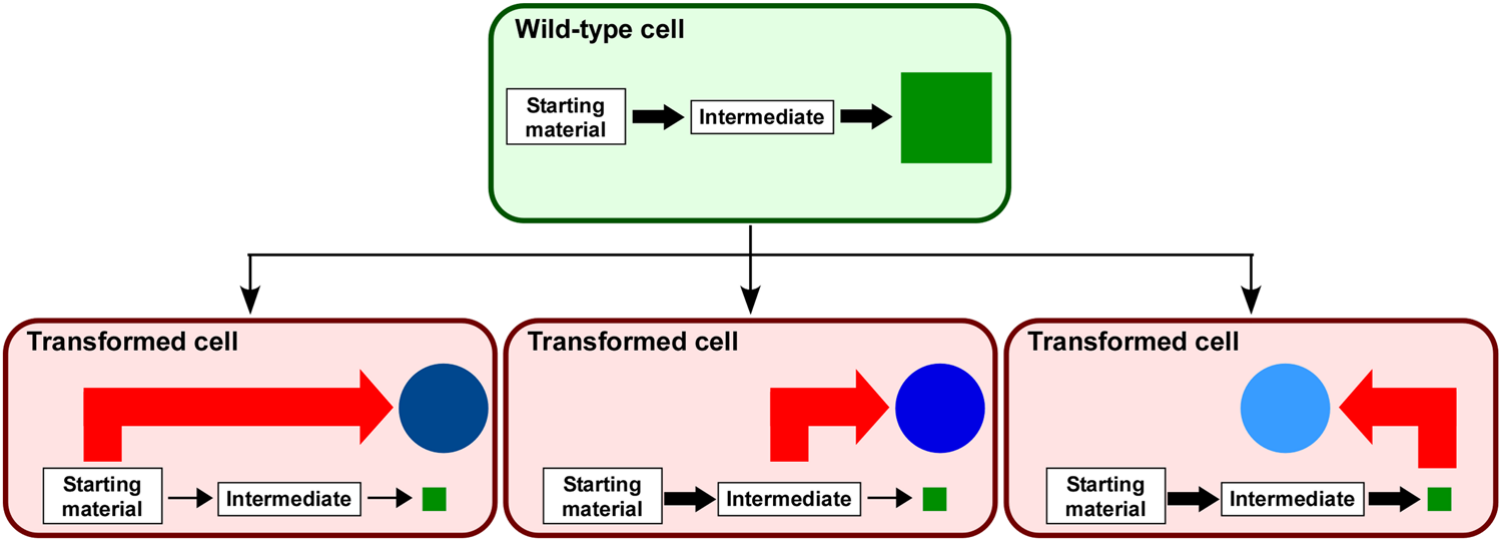
Schematic representation of “rational metabolic-flow switching”. The first step is the identification of a major secondary metabolite (filled green square) in the wild-type cells, which are the intended host cells, and the metabolite indicates a specialised active biosynthetic pathway in the cells. The second step is the introduction of an exogenous biosynthetic enzyme (red arrow) utilising the abundantly present starting material, intermediate, or the end-product of the original secondary metabolic pathway, which allows for the efficient switching of the pathway to the production of the exogenous secondary metabolite (filled blue circle), which has the same skeleton as the major compound that was originally present in the host cells.

Here, we demonstrated the usefulness of “rational metabolic-flow switching” for the efficient production of exogenous secondary metabolites using plant cells as the host. The strategy involves two steps (Fig. 6). First, a major secondary metabolite is identified that indicates the active metabolic pathway(s) in the host plant cells under specific culture conditions. Second, the cells are genetically transformed to express exogenous biosynthetic enzyme(s) required to form a new biosynthetic pathway for the target compound, which branches off from the starting material, intermediate, or the end-product of the active biosynthetic pathway identified in the first step. The most important points are to assess the eligibility of each plant cell host and to take advantage of the active biosynthetic pathway that is inherent in the host cells. The metabolic engineering of plant cell cultures has focused more on simply activating the existing pathways, such as overexpressing a rate-limiting biosynthetic enzyme, but such approaches do not necessarily lead to the increased accumulation of desired end-products^21^. The strategy we proposed here is an alternative metabolic engineering strategy that switches the existing biosynthetic pathway to a new pathway, and it should work well compared with the conventional strategy.

Subcellular localisation of the exogenous biosynthetic enzyme to be introduced into the host cells should also be taken into consideration for the efficient substrate supply to the introduced enzyme. The acyltransferase catalysing FP formation from feruloyl-CoA and putrescine in the Pn cells, which we identified recently, is the cytosolic enzyme (unpublished data), like other BAHD family acyltransferases^28^, including the ACT^29^, which means that feruloyl-CoA and putrescine are sufficiently present in cytosol. It is considered that the successful production of pCA in the ACT transformants was achieved, because the cytosolic ACT enzyme could use *p*-coumaroyl-CoA and agmatine, which are supplied abundantly to cytosol following their biosynthesis in cytosol and plastids, respectively, like feruloyl-CoA and putrescine^47–49^. Matching the subcellular localisation of inherently present substrate(s) and exogenously introduced enzyme is the key for successful production of desired compound.

The present study demonstrated that the Pn cells are excellent hosts for the production of phenylpropanoid compounds. Because of their antitumor, antioxidant, antiviral, and anti-inflammatory properties, phenylpropanoid compounds have been the focus of intensive research^15^. However, most of the phenylpropanoid compounds currently used in medicinal and cosmetic applications are extracted from native plant sources. Therefore, the compounds that are present abundantly are used but not the minor constituents, regardless of whether they have superior properties. Thus, there has been much interest in developing novel biosynthetic techniques to produce useful phenylpropanoids in an efficient and cost-effective manner^15^. To construct phenylpropanoid biosynthetic pathways in microbial hosts, it is essential to introduce enzymes for very early biosynthetic reactions, which leads to the formation of the key intermediate *p*-coumaroyl-CoA from Phe^17^. In contrast, judging from the efficient production of pCA in the ACT transformed Pn cells, *p*-coumaroyl-CoA is supplied abundantly in an accessible form for the exogenous enzyme without any genetic manipulation. This biosynthetic property of Pn cells provides a great advantage over the microbial hosts in the establishment of a phenylpropanoid-producing system.

By developing a series of host plant cells and culture conditions with distinct secondary metabolic properties, the versatility of the “rational metabolic-flow switching” would be expanded and could be applied to the production of a variety of high-value compounds, such as terpenoids and alkaloids, using the plant cells as production hosts. At present, only 14 substances or products are produced commercially from plant cell cultures^19^, which indicates that most plant cell culture systems established over many decades of research have not met the requirements to produce desired compound. As long as a certain secondary metabolic pathway is active (even if the desired compound is not accumulated) and a stable transformation system is available, such cell cultures may be used as production hosts for exogenous secondary metabolites by applying the “rational metabolic-flow switching” strategy. This will lead to the revival of unused and under-used cell cultures and expand the use of plant cell cultures as producers of high-value compounds.

## Electronic supplementary material

Supplementary information

## Data Availability

All data generated or analysed during this study are available from the corresponding author on reasonable request.
