# Supplementary material for: Rational metabolic-flow switching for the production of exogenous secondary metabolites in bamboo suspension cells
Source: Sci Rep. 2018 Sep 4;8:13203. doi: 10.1038/s41598-018-31566-4 (PMC6123407; doi:10.1038/s41598-018-31566-4)
Supplement: Supplementary file 1 — Supplementary information [file 41598_2018_31566_MOESM1_ESM.pdf]

Supplementary Information

for:

Rational metabolic-flow switching for the production of exogenous secondary metabolites in bamboo suspension cells

Taiji Nomura[1,*], Shinjiro Ogita[2], Yasuo Kato[1]

[1]*Biotechnology Research Center and Department of Biotechnology, Toyama Prefectural University, 5180 Kurokawa, Imizu, Toyama 939-0398, Japan*

[2]*Faculty of Life and Environmental Sciences, Prefectural University of Hiroshima, 5562 Nanatsukacho, Shobara, Hiroshima 727-0023, Japan*

[*]Corresponding author. e-mail: tnomura@pu-toyama.ac.jp

Supplementary Methods

Genomic and RT-PCR analyses

PCR analyses for verification of the presence and transcription of full-length *HvACT1* and *HPT* genes in the transformed Pn cells were performed using three sets of gene-specific primers:

for *HvACT1* (full-length coding sequence),

5′-ATGAAGATCACCGTGCACTC-3′ (forward) and

5′-CTAGTCGAGGCTGTAGCAGCAG-3′ (reverse);

for *HPT* (full-length coding sequence),

5′-ATGAAAAAGCCTGAACTCACC-3′ (forward) and

5′-CTATTCCTTTGCCCTCGGAC-3′ (reverse);

for actin (endogenous standard, partial coding sequence),

5′-GGTATACGCTTCCTCACGCT-3′ (forward) and

5′-CTCGCAGTCTCAAGCTCCT-3′ (reverse).

The genomic PCR reactions were performed in 20-μL reaction mixture containing 50 ng of genomic DNA, 0.5 μM each primer, 0.2 mM dNTPs, 1× reaction buffer, and 0.5 U of Blend *Taq* DNA polymerase (Toyobo, Osaka, Japan). After a 2-min incubation at 94°C, 35 cycles were performed as follows: for *HvACT1*, 30 s at 96°C, 30 s at 64°C, and 1 min at 72°C; for *HPT*, 30 s at 96°C, 30 s at 68°C, and 1 min at 72°C; and for actin, 30 s at 96°C and 1 min at 68°C. For the RT-PCR analysis, the PCR reactions were performed as for the genomic PCR, except 50 ng of synthesised cDNA was used as the template, 1.25 U of Blend *Taq* DNA polymerase was used, and the amplification cycles were increased to 40 for the *HvACT1* and *HPT* genes. The amplified products were detected with ethidium bromide staining after agarose-gel electrophoresis.

RT-PCR analysis for *HvACT1* transcription in the ACT transformants during 16-d culture period was performed using gene-specific primers,

5′-ACCGGCAACGTCATCCTCTG-3′ (forward) and

5′-CTAGTCGAGGCTGTAGCAGCAG-3′ (reverse),

which amplify the partial coding sequence. The PCR reactions were performed in 20-μL reaction mixture containing 50 ng of cDNA, 0.5 μM each primer, 0.2 mM dNTPs, 1× reaction buffer, and 0.5 U of Blend *Taq* DNA polymerase. After a 2-min incubation at 94°C, 35 cycles were performed as follows: 30 s at 96°C, 30 s at 68°C, and 1 min at 72°C. The amplified products were detected with ethidium bromide staining after agarose-gel electrophoresis.

Expression and purification of recombinant HvACT1 enzyme

The pUC118 plasmid harbouring *HvACT1* cDNA[1] was used as the PCR template to prepare DNA encoding the full-length HvACT1 enzyme. The DNA fragment was amplified with the primers,

5′-CGCGCGGCAGCCATATGAAGATCACCGTGCACTCTTC-3′ (forward) and

5′-GTCATGCTAGCCATACTAGTCGAGGCTGTAGCAG-3′ (reverse).

The amplified DNA was inserted into the NdeI site of vector pET28a (Novagen, Madison, WI, USA) using an In-Fusion HD Cloning Kit (Takara Bio). The resulting plasmid was introduced into *Escherichia coli* strain BL21-CodonPlus (DE3)-RIL (Agilent Technologies, Santa Clara, CA, USA) for expression of the N-terminally His-tagged enzyme.

Culturing of the recombinant *E. coli*, induction of enzyme expression, and the extraction and purification of the His-tag-free recombinant enzyme were performed as described previously[2] except for the use of 50 mM Tris-HCl buffer (pH 7.5) as the basal buffer.

Table S1 Specific activity levels of the recombinant HvACT1 enzyme.

Substrate[a]		Specific activity	Relative activity
Acyl-donor	Acyl-acceptor	(nkat/mg)[b]	(%)
p-Coumaroyl-CoA	Agmatine	870 ± 35	100
Feruloyl-CoA	Agmatine	480 ± 1.7	55
p-Coumaroyl-CoA	Putrescine	3.3 ± 0.11	0.38
Feruloyl-CoA	Putrescine	1.0 ± 0.014	0.11

Data are means ± SDs (*n* = 3).

[a]Enzyme activity was measured at 100 μM.

[b]One katal (kat) of enzyme activity was defined as the amount of enzyme that catalyses the formation of the reaction product at a rate of 1 mol/s.

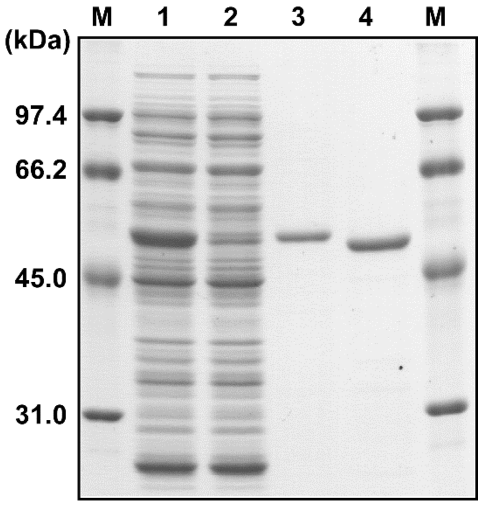

Figure S1 Purification of the recombinant HvACT1 enzyme expressed in *E. coli*.

Protein from each purification step was separated by SDS-PAGE on a 12.5% gel and stained with Coomassie Brilliant Blue R-250. Lane M, Molecular size markers; Lane 1, crude *E. coli* extract; Lane 2, column run-through from metal-affinity chromatography; Lane 3, eluate from metal-affinity chromatography; Lane 4, Superdex 200 after thrombin digestion.

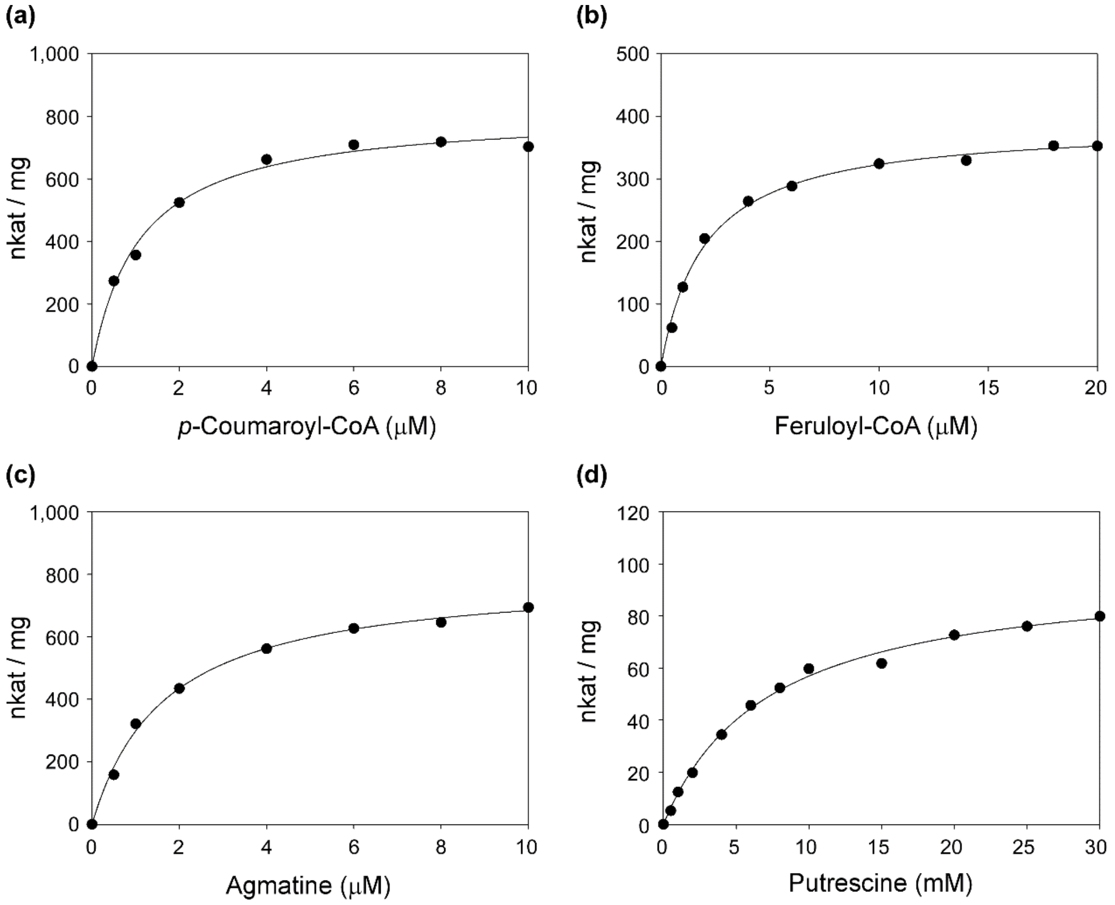

Figure S2 Michaelis–Menten plots of the recombinant HvACT1 enzyme expressed in *E. coli* for acyl-donors (a, b) and acyl-acceptors (c, d).

(**a**) Dependence of pCA formation on the concentration of *p*-coumaroyl-CoA in the presence of 100 μM agmatine, (**b**) dependence of FA formation on the concentration of feruloyl-CoA in the presence of 100 μM agmatine, (**c**) dependence of pCA formation on the concentration of agmatine in the presence of 100 μM *p*-coumaroyl-CoA, (**d**) dependence of pCP formation on the concentration of putrescine in the presence of 100 μM *p*-coumaroyl-CoA. n = 1. One katal (kat) of enzyme activity was defined as the amount of enzyme that catalyses the formation of the reaction product at a rate of 1 mol/s. Correlation coefficients (R^2) in the nonlinear regressions in (**a**), (**b**), (**c**), and (**d**) were 0.993, 0.996, 0.997, and 0.996, respectively. See Table 1 for the kinetic parameters as calculated by nonlinear regression of the data to the Michaelis–Menten equation.

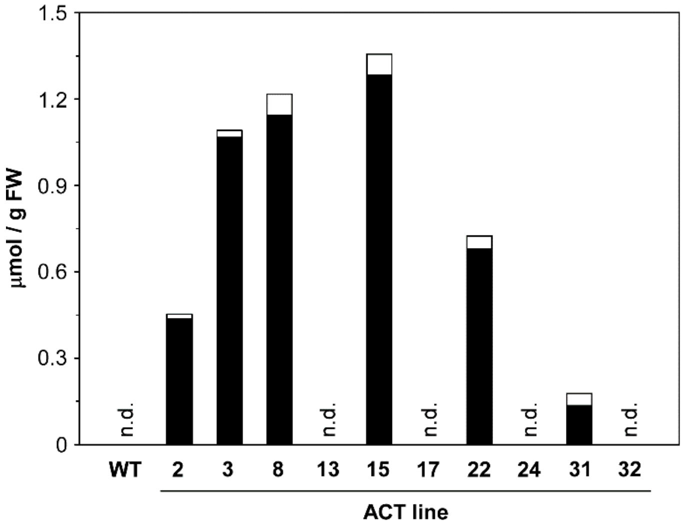

Figure S3 The pCA and FA contents in 10 calli selected as potential ACT transformants.

Two-week-old calli grown on a solid selective medium were analysed (n = 1). ■, pCA; □, FA; WT, wild-type; n.d., not detected.

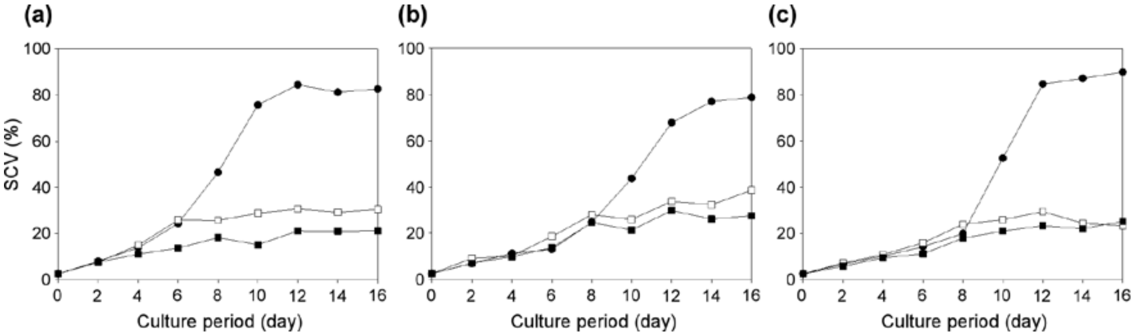

Figure S4 Growth profiles of ACT transformed suspension cells.

Growth profiles of suspension cells starting from 2.5% SCV (sedimented cell volume per 100 mL medium) under PR (●), LG1 (□), and LG2 (■) conditions. (**a**) Wild-type, (**b**) ACT-line 15, (**c**) ACT-line 22. $n = 1$.

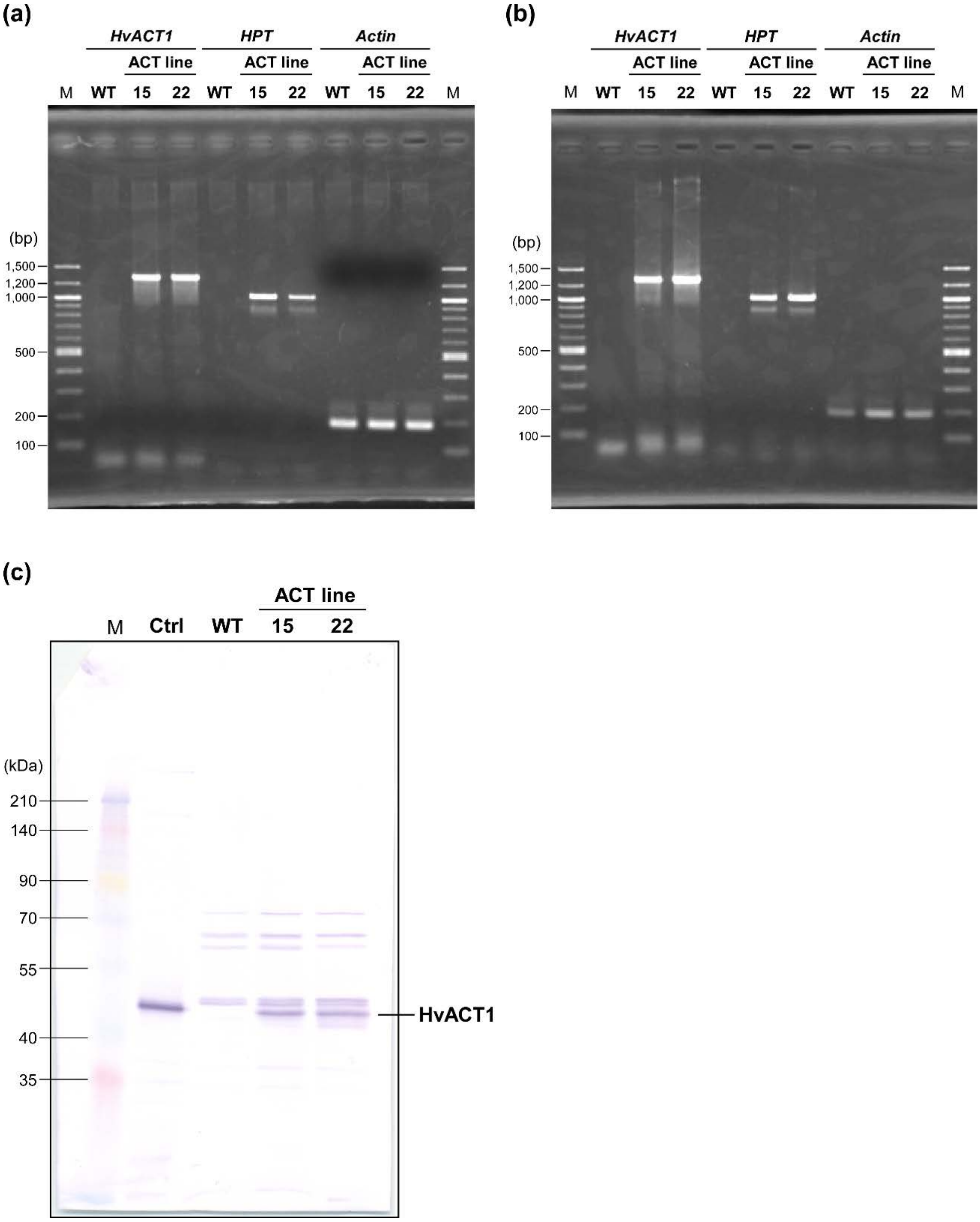

Figure S5 Full-length agarose gels and blot for the data presented in Fig. 2.

(**a**) Genomic PCR gel, (**b**) RT-PCR gel, (**c**) immunoblot. Lane M in (**a**) and (**b**) is 100 bp DNA ladder marker (New England BioLabs, Ipswich, MA, USA) and that in (**c**) is prestained protein XL-ladder marker (APRO Life Science Institute).

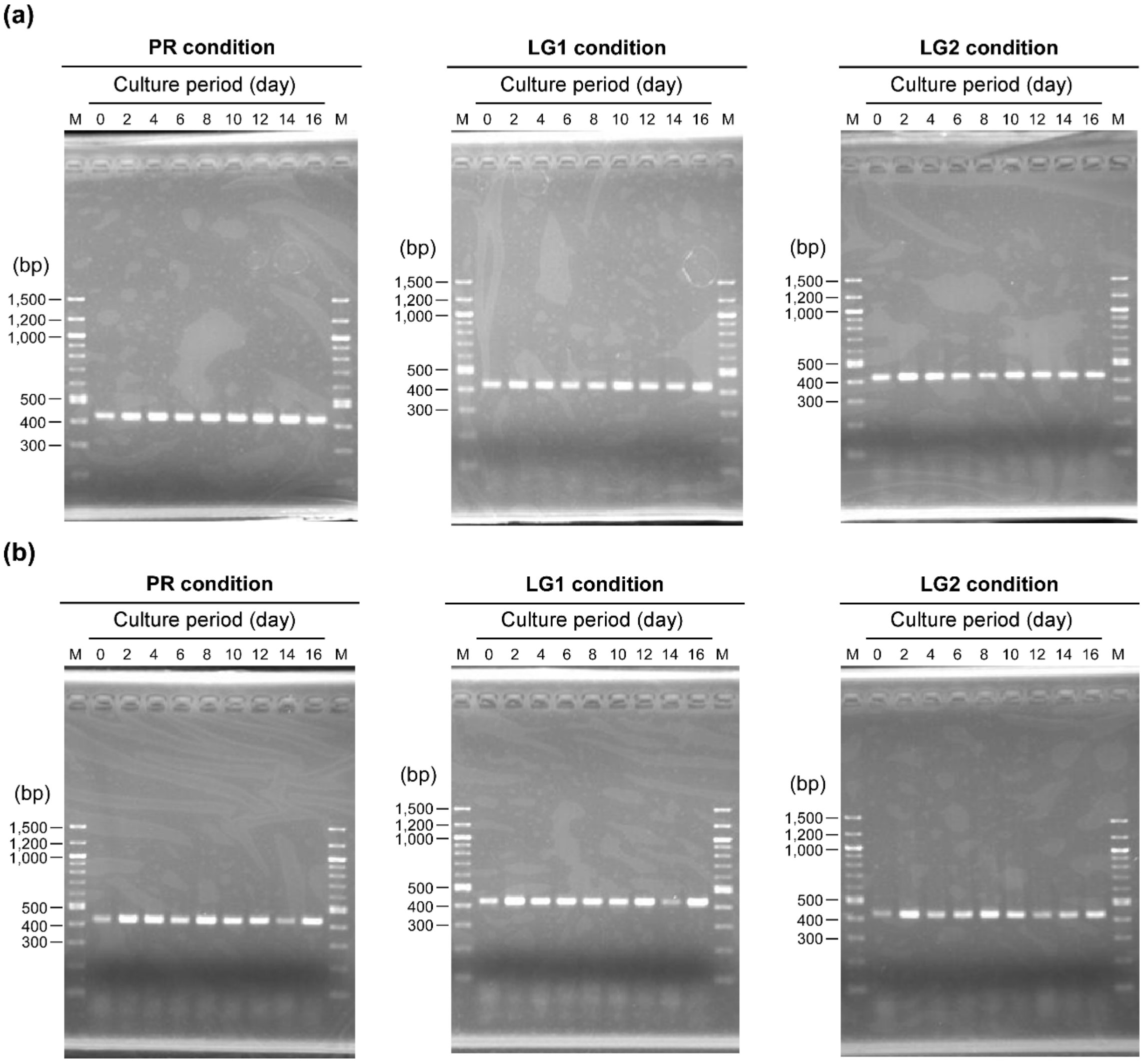

Figure S6 Full-length agarose gels for the data presented in Fig. 3.

(**a**) ACT-line 15, (**b**) ACT-line 22. Lane M, 100 bp DNA ladder marker.

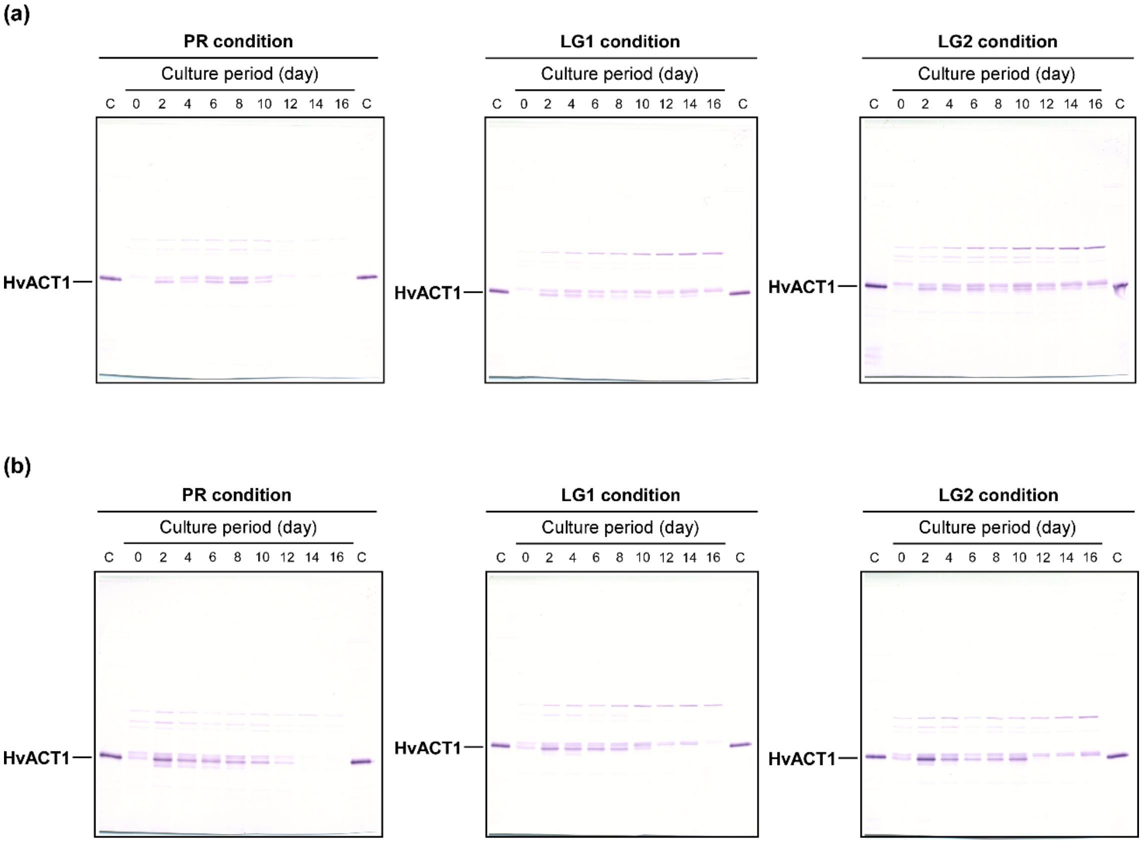

Figure S7 Full-length blots for the data presented in Fig. 3.

(**a**) ACT-line 15, (**b**) ACT-line 22. Lane C is a positive control (recombinant His-tag-free HvACT1 enzyme expressed in *E. coli*).

References for Supplementary Information

1. Nomura, T. *et al*. Chromosome arm location of the genes for the biosynthesis of hordatines in barley. *Genes Genet. Syst.* **82**, 455–464 (2007).
2. Nomura, T., Tsuchigami, A., Ogita, S. & Kato, Y. Molecular diversity of tuliposide A-converting enzyme in the tulip. *Biosci. Biotechnol. Biochem.* **77**, 1042–1048 (2013).
